# Supplementary figures and images for: PGRP-LA regulates peritrophic matrix synthesis and influences trypanosome infection outcomes in tsetse flies
Source: PLoS Pathog. 2026 May 4;22(5):e1013520. doi: 10.1371/journal.ppat.1013520 (PMC13160434; doi:10.1371/journal.ppat.1013520)

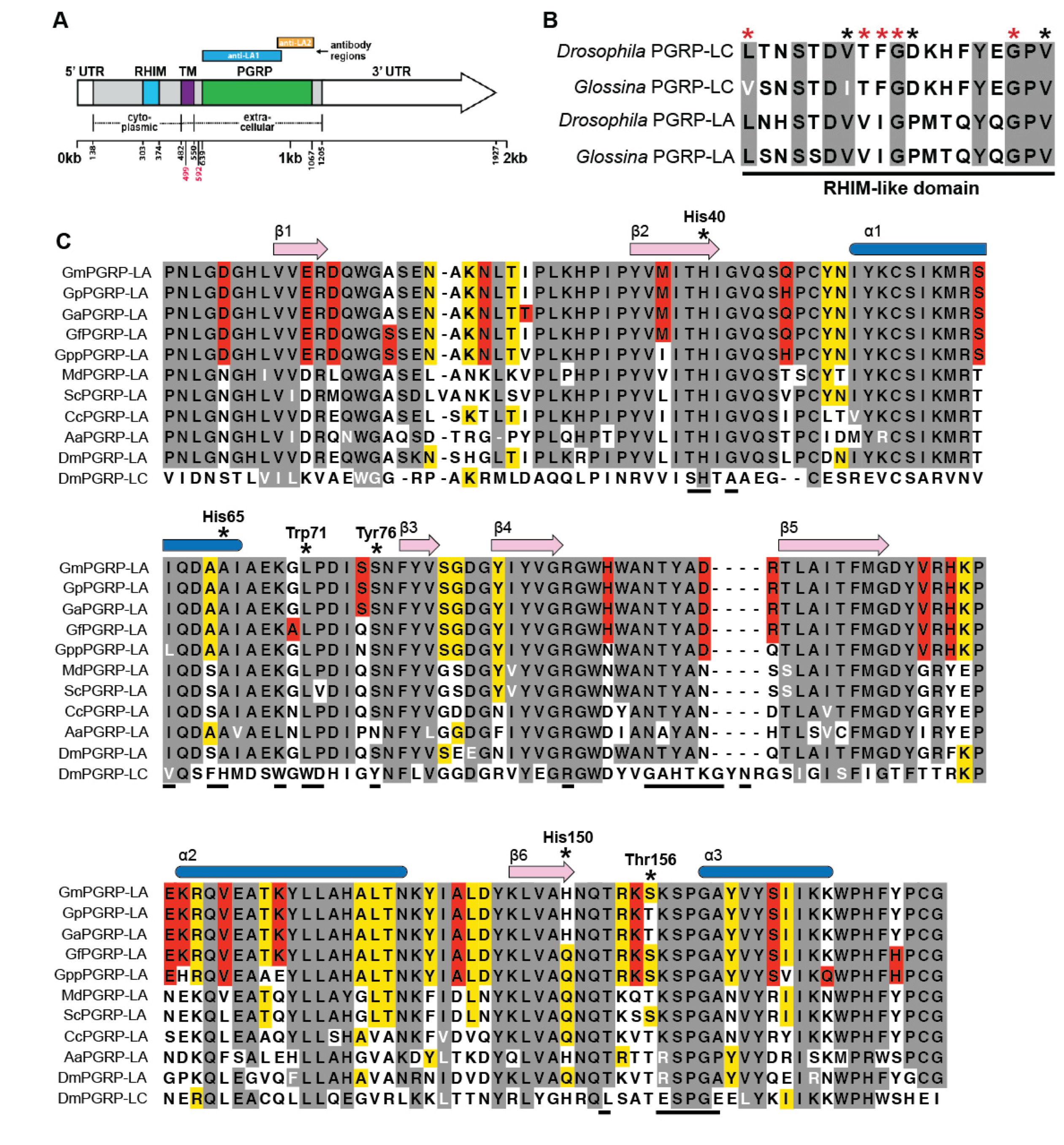

Supplement: S1 Fig — (A) Schematic view of pgrp-la and its putative product. The putative product contains an extracellular PGRP domain (green), a transmembrane domain (TM, dark purple) and a cytoplasmic region with a RHIM-like domain (blue). Coding sequence (CDS) regions lacking known functional domains are shown in gray. No signal peptide was predicted. The regions used to generate the recombinant proteins for antibody production (anti-LA1 and anti-LA2) are also indicated. (B) Protein sequence alignment of the RHIM-like domain from Glossina morsitans (Gm) PGRP-LC and PGRP-LA, and Drosophila melanogaster (Dm) PGRP-LC and PGRP-LA. Residues marked with red asterisk are essential for DmPGRP-LC RHIM-like domain to activate IMD pathway, while black asterisk are contributing to the activation without being mandatory. The grey shaded amino acids depict conserved or similar residues written in black and white, respectively. (C) Protein sequence alignment of the PGRP domain of PGRP-LA from G. morsitans morsitans (Gm), G. pallidipes (Gp), G. austeni (Ga), G. fuscipes fuscipes (Gf), G. p. palpalis (Gpp), Musca domestica (Md), Stomoxys calcitrans (Sc), Ceratitis capitata (Cc), Aedes aegypti (Aa) and D. melanogaster (Dm). PGRP domain from DmPGRP-LC is presented as a reference. Residues interacting with bacterial PGN in DmPGRP-LC, which are based on the crystal structure of Drosophila PGRP-LC [44] are underlined in black at the bottom of the alignment. Asterisks indicate amino acids required for amidase activity (numbering starts at the beginning of the PGRP domain). Alpha helices (α) and Beta strands (β) are represented in blue and pink, respectively. Grey background displays conserved amino acids with identities and similarities written in black and white, respectively. Red shaded residues indicate Glossina specific substitution. Yellow shading indicates residues conserved in Glossina plus another taxa. (TIFF) [file ppat.1013520.s001.tiff]

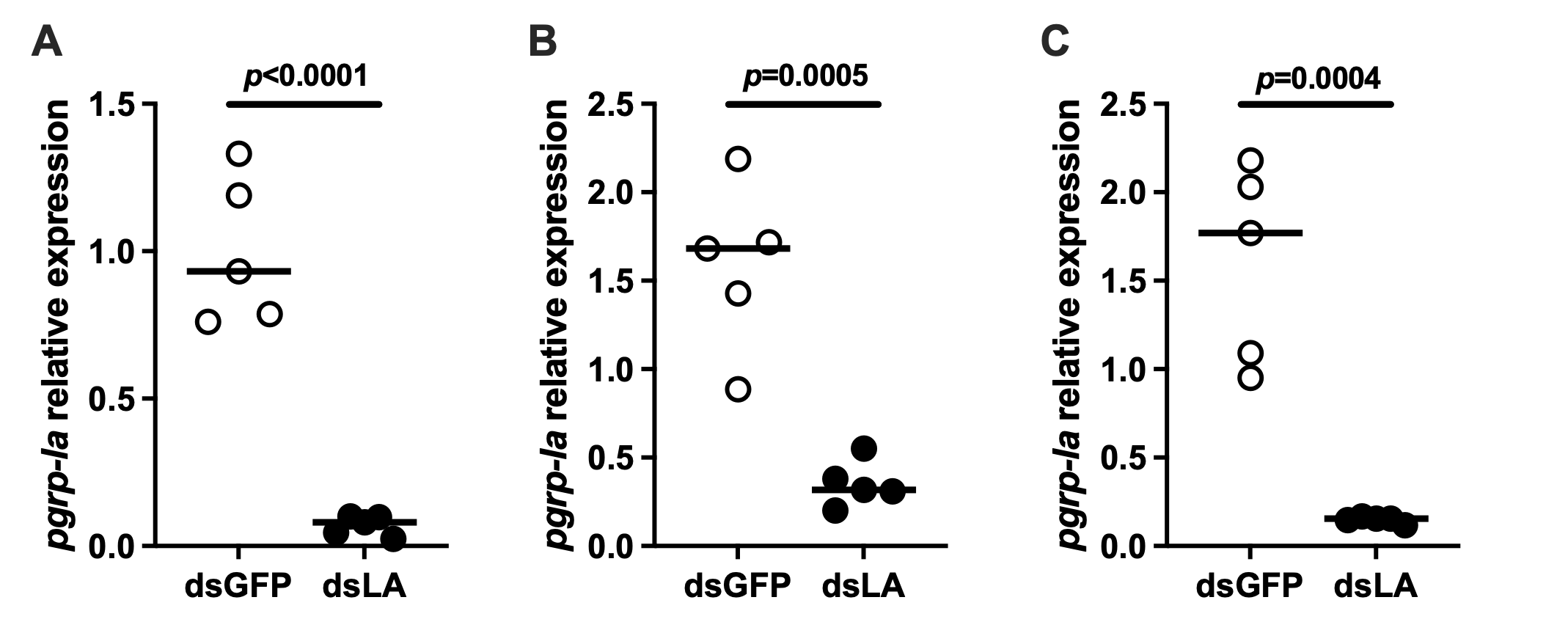

Supplement: S2 Fig — Relative pgrp-la expression in cardia of dsLA (treatment) and dsGFP (control) treated flies prior to challenge with (A) trypanosomes, (B) E. coli, and (C) Serratia. Each dot on the graph represents one biological replicate, each replicate containing cardia from five dsRNA treated individuals. Statistical significance was determined via student’s t-test (GraphPad Prism v.10.4.1). (TIFF) [file ppat.1013520.s002.tiff]

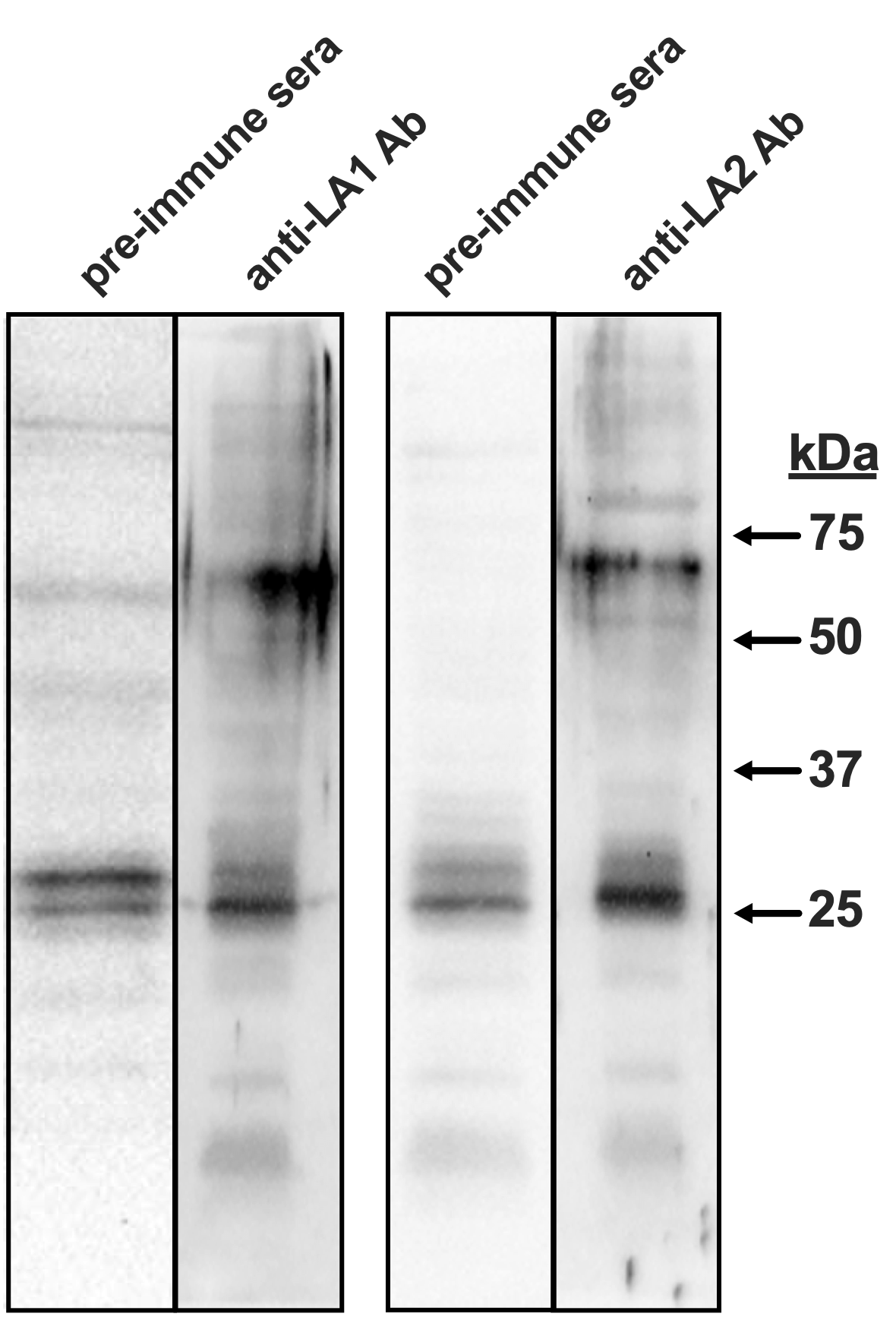

Supplement: S3 Fig — Western blot analysis was performed using protein extracts from cardia of 5 individual flies. Blots were incubated overnight at 4°C with either pre-immune sera or with either anti-LA1 or anti-LA2 antibodies (both diluted at 1:10,000), exposed to HRP-conjugated anti-rabbit secondary antibody (diluted at 1:20,000) and visualized using a SuperSignal West Pico Chemiluminescent Substrate kit. The protein size standards are indicated. (TIFF) [file ppat.1013520.s003.tiff]
